# Impaired Glutathione Redox System Paradoxically Suppresses Angiotensin II-Induced Vascular Remodeling

**DOI:** 10.1371/journal.pone.0108115

**Published:** 2014-10-24

**Authors:** Kazuma Izawa, Motoi Okada, Kazuhiro Sumitomo, Naoki Nakagawa, Yoshiaki Aizawa, Junichi Kawabe, Kenjiro Kikuchi, Naoyuki Hasebe

**Affiliations:** Division of Cardiology, Nephrology, Pulmonology and Neurology, Department of Internal Medicine, Asahikawa Medical University, Asahikawa, Japan; Faculty of Medicine & Health Sciences, United Arab Emirates

## Abstract

**Background:**

Angiotensin II (AII) plays a central role in vascular remodeling via oxidative stress. However, the interaction between AII and reduced glutathione (GSH) redox status in cardiovascular remodeling remains unknown.

**Methods:**

*In vivo:* The cuff-induced vascular injury model was applied to Sprague Dawley rats. Then we administered saline or a GSH inhibitor, buthionine sulfoximine (BSO, 30 mmol/L in drinking water) for a week, subsequently administered 4 more weeks by osmotic pump with saline or AII (200 ng/kg/minute) to the rats. *In vitro:* Incorporation of bromodeoxyuridine (BrdU) was measured to determine DNA synthesis in cultured rat vascular smooth muscle cells (VSMCs).

**Results:**

BSO reduced whole blood GSH levels. Systolic blood pressure was increased up to 215±4 mmHg by AII at 4 weeks (p<0.01), which was not affected by BSO. Superoxide production in vascular wall was increased by AII and BSO alone, and was markedly enhanced by AII+BSO. The left ventricular weight to body weight ratio was significantly increased in AII and AII+BSO as compared to controls (2.52±0.08, 2.50±0.09 and 2.10±0.07 mg/g respectively, p<0.05). Surprisingly, the co-treatment of BSO totally abolished these morphological changes. Although the vascular circumferential wall stress was well compensated in AII, significantly increased in AII+BSO. The anti-single-stranded DNA staining revealed increasing apoptotic cells in the neointima of injured arteries in BSO groups. BrdU incorporation in cultured VSMCs with AII was increased dose-dependently. Furthermore it was totally abolished by BSO and was reversed by GSH monoethyl ester.

**Conclusions:**

We demonstrated that a vast oxidative stress in impaired GSH redox system totally abolished AII-induced vascular, not cardiac remodeling via enhancement of apoptosis in the neointima and suppression of cell growth in the media. The drastic suppression of remodeling may result in fragile vasculature intolerable to mechanical stress by AII.

## Introduction

Increased generation of reactive oxygen species (ROS) and/or depletion of antioxidant capacity lead to enhanced ROS activity and oxidative stress. In certain pathological conditions, such as hypertension, diabetes mellitus and arteriosclerosis, oxidative stress is considered to be a significant contributor to vascular remodeling [1], [2].

Considerable evidence implicates angiotensin II (AII) is one of the key factors of vascular remodeling [3]. Chronic infusion of AII induces the cardiac hypertrophy and marked vascular remodeling [4]. AII reportedly induces vascular smooth muscle cell (VSMC) hypertrophy, proliferation, and migration via ROS generation by stimulation of NAD(P)H oxidase [5], [6]. AII is also a potent trigger of apoptosis [7], [8]. Apoptosis plays a potential compensatory role by countervailing mechanism against cell growth. We have reported that enhanced apoptotic mechanism prevents neointimal thickness in a balloon injured model [9]. Both cell growth and apoptosis are considered to be the major mechanisms that have been invoked to contribute to vascular remodeling by AII. It is not known, however, whether both mechanisms in vascular remodeling are equally facilitated under the condition with enhanced oxidative stress. If both mechanisms could be equally facilitated, it is noteworthy to understand what modification is born in the phenotype of AII-induced vascular remodeling with enhanced oxidative stress.

In the present study, we revealed GSH redox system on vasculature. In order to enhance oxdative stress in vivo, we utilized not only AII, but also buthionine sulfoximine (BSO), a selective inhibitor of γ–glutamylcysteine synthase, an enzyme of the glutathione (GSH) biosynthesis pathway [10]. In fact, BSO administration is known to enhance oxidative stress by reducing the tissue GSH level [11]–[14].

The primary goal of the present study was to investigate AII-induced vascular remodeling under the condition of enhanced oxidative stress. To accomplish this goal we applied a chronic infusion of AII in rat cuff injury model [8], [15]–[17], with or without BSO-induced impairment of GSH redox system. The rat cuff injury model, which was treated by a surgically placed hollow polyethylene tube around femoral artery, is known to produce a diffuse intimal thickness of artery that is similar to early lesions of atherosclerosis [16].

## Materials and Methods

### Animals and Treatment

All animal work described in this study was carried out according to the guidelines of the Ethics Committee on Laboratory Animals of Asahikawa Medical University, and this study was specifically approved by the Ethics Committee.

Male Sprague-Dawley rat (9–10 weeks of age with an average weight of 297 g; Charles River Co, Tokyo, Japan) were used in this study. Rats were placed on a 12-hour-day/night cycle at 22–24°C. After 1-week-acclimatization, rats were used for the following experiments. Arterial blood pressure and heart rate (HR) were measured by a tai-cuff method (BP-98A, Softron Co, Tokyo, Japan). The intake of water and body weight (BW) were continuously monitored during the experiment. Rats were separated into two groups; orally received with either glutathione synthase inhibitor buthionine sulfoximine (BSO, 30 mmol/L in drinking water, BSO group n = 14) or its vehicle solution for 5 weeks (n = 14). At 1 week of treatment, we made the vascular injury model. Polyethylene cuff (length 6–8 mm Becton Dickinson and Company, NJ, USA) was placed loosely around the right femoral artery as described previously [14], then we divided each group into two more groups, either receiving AII (200 ng/kg per minute s.c. AII; n = 7, AII+BSO; n = 7) or its vehicle using osmotic pump (Control; n = 7, BSO; n = 7). After 4 weeks of vascular injury, rats were killed and collected their blood samples, hearts, aortas and femoral arteries.

### Measurement of Plasma GSH and Whole Blood GSH/GSSG

Collected plasma samples were treated with 0.5 mol/L percholic acid and 100 µmol/L EDTA, followed by centrifugation at 12000 rpm for 10 minutes. The supernatants were applied to HPLC to measure the amount of GSH as described before [18]. The amount of GSH/GSSG in blood sample was determined by the previously described method [19] with some modification. Briefly, whole blood was mixed with 4% 5-sulfosalicylic acid (SSA) and centrifuged at 3500 rpm for 5 minutes at 4°C. The supernatants were stored at −85°C for further use. The solutions were mixed with 5-volume of 5% SSA. GSH content in all samples was measured in the supernatant by the GSSG reductase recycling assay utilizing with 5, 5-dithiobis-2-nitrobenzoic acid. The colored product is read at 405 nm and quantitated using a standard curve.

### Measurement of intracellular Reactive Oxygen Species generation in Neutrophil

The level of reactive oxygen species (ROS) in neutrophils were quantitated by the 10 µmol/L 2′, 7′-dichlorodihydrofluorescein diacetate (CDCFH-DA) bis (acetoxymethyl) ester (Molecular Probes) method, based on the ROS-dependent oxidation of CDCFH-DA to CDCFH as described previously [20]. White blood cells (WBCs) were separated by Ficoll density gradient method. The residual WBCs were washed with (10 mmol/L) HEPES solution twice. And incubated in HEPES containing CDCFH of 10 µmol/L for 15 minutes, exposed another 15 minutes with or without 10 nmol/L of PMA (Phorbol 12-Myristate 13-Acetate Sigma). ROS production in polymorph nuclear cells (PMNCs) was measured by flow-cytometer (Beckman Coulter). The fluorescence of cells was recorded under 488 nm excitation. ROS production was quantified by mean DCFH fluorescence intensities.

### Superoxide detection in arterial sections

The levels of superoxide (**^·^**O_2_ **^−^**) production in the aortas were detected by the method as described before [21]–[23]. Briefly, we freshly isolated the left femoral arteries and cuff injured right femoral arteries, fixed in OCT compound and made frozen section. The 30-µm-thick arterial sections were incubated with dihydroethidium (DHE) (10 µmol/L) at 37°C for 30 minutes. DHE binds with superoxide to form 2-hydroxyethidium which can be imaged using confocal microscopy (Fluoview FV1000, Olympus). Detection wavelength settings was 575–700 nm for DHE (red) imaging.

Then we calculated DHE signal/auto fluorescence signal ratio and compared each group using NIH image-J analysis software (NIH Research Service Branch). All procedures were carried out under shading.

### Measurement of Vascular Wall Thickness

To measure thickness on intimal and medial layers of cuff injured arteries, 3 cross sections of each artery spaced at 1 mm intervals were stained with hematoxylin and eosin. The cross-sectional intimal and medial areas of each section in a given photomicrograph were determined with NIH Image-J. Then, the ratio of average intimal area to medial area was calculated for each artery [14] The medial wall thickness of aortas and coronary arteries was also assessed as the ratio of thickness of media to the outer radius of vessels. Values were corrected to the radius of arteries, which were assumed to be circular, by the following calculation: Wall thickness ratio = (R-r)/R, where R is the radius of the outer circumference, and R-r represents medial thickness [24].

### Measurements of Apoptosis

Arterial sections were incubated with rabbit anti-single-stranded DNA (ssDNA) [9], [25] polyclonal Rabbit antibody (Dako Japan Co Ltd) and mouse anti-proliferating cell nuclear antigen (PCNA; Dako Co) at 4°C for overnight. The signal was visualized by peroxidase-conjugated antibody and 3, 3′diaminobenzidine (DAB; DAKO Co). Nuclei were counterstained with hematoxylin. To quantify apoptosis, the percentage of apoptotic cells in the total cell population was calculated by counting all cells from 4 random microscopic fields at a magnification of ×200.

### Cell Culture and Treatments

Rat VSMCs were prepared from the thoracic aorta of 5week-old male Sprague-Dawley rats by explant technique [9], [26]. Cells were cultured in DMEM containing 10% fetal bovine serum (FBS), and were grown to subconfluence and made quiescent by incubation in FBS free DMEM for 24 hours before use. Cells were pretreated with BSO (0, 1, 10 and 100 µmol/L) for 12 hours with or without GSH mono-ethyl ester (1 mmol/L), and AII (0.1 µmol/L SIGMA Chemical Co. USA) was added during the last 12 hours. For all experiments, passages 3 to 6 of subcultured cells were used.

### VSMC GSH Content

VSMCs were plated on 35 mm culture dishes and treated with or without BSO. Dishes were then washed three times with PBS (calcium and magnesium free) and VSMCs were collected.GSH content were measured as HPLC method as described above [18].

### Cell proliferation

BrdU Cell Proliferation Assay kit (Exalpha Biologicals, Inc.) were used to quantify cell proliferation. BrdU was added another last 4 hours. Values of BrdU assay in no treated control cells were set at 100%.

### Circumferential Vascular Wall Stress

Circumferential vascular wall stress (σ_c_) was calculated from the law of Laplace (in units of force per unit area) as σ_c_ = P⋅r/w, where P is pressure, r is the lumen radius of the artery, and w is the wall thickness. We measured σ_c_ in regard to both mean (σ_cMAP_) and systolic blood pressures (σ_cSAP_).

### Expression of NADPH oxidase

We performed another series of experiments on cuff injured model rats in order to study the role of NADPH oxidase. We used additional 20 cuff injury model rats, divided into 4 groups (no drug administration control, BSO, AII and AII with BSO, n = 5 of each group) and were sacrificed at 1 week after drug administration. Arterial sections were incubated with rabbit polyclonal anti-goat gp91-phox (Santa Cruz Biotechnology, 1∶100) at 4°C for overnight. The signal was visualized by peroxidase-conjugated antibody and 3, 3′diaminobenzidine (DAB; DAKO Co). To quantify the NADPH oxidase expression, the percentage of gp91-phox positive cells in the total cell population in adventitia was calculated by counting all cells from 4 random microscopic fields at a magnification of ×200.

### Statistical Analysis

All results are expressed as mean ± SEM. Statistical comparisons were evaluated using one-way ANOVA followed by Bonferroni post hoc analysis. A probability value of *p*<0.05 was considered statistically significant.

## Results

### Blood Pressure, Heart Rate, LV to Body Weight Ratio

There was no difference in the amount of water intake among groups. An average intake of BSO was 3.13 mmol/kg/day (n = 14). AII-treatment significantly increased blood pressure, but there were no significant differences with or without BSO treatments (Table 1). LV to body weight ratio was significantly increased by AII stimulation, however it was not significantly affected by BSO (Table 1). Body weight and heart rate were no significant difference between 4 groups (Table 1).

**Table 1:** Body weight, Heart rate, Left Ventricular Weight/Body Weight Ratio, Whole Blood GSH/GSSG Concentration, Plasma GSH Concentration.

	Body weight (g)	LV wt/body wt (mg/g)	Systolic BP (mmHg)	Heart rate (/min)	Whole blood (GSH mmol/L)	Whole blood (GSSG mmol/L)	Plasma GSH (mmol/L)
Control	405.0±10.8	2.10±0.07	106.0±2.9	304.8±5.5	1.89±0.13	0.19±0.02	4.37±0.48
BSO	389.2±6.9	2.13±0.05	115.3±3.0	313.2±4.7	1.23±0.05**††	0.09±0.02 *†	1.78±0.26 **
AII	402.8±6.8	2.52±0.08 *	214.6±4.1 **	331.0±13.6	2.12±0.10	0.17±0.03	2.46±0.33*
AII+BSO	388.4±11.7	2.50±0.09 *	212.4±6.6 **	296.1±8.1	1.42±0.10*††	0.09±0.02 *†	1.88±0.36 **

BSO: buthionine sulfoximine (30 mmol/L in drinking water) treated group, AII:angiotensin II (200 ng/minute/kg osmotic pump) treated group, AII+BSO:angiotensin II and BSO co-treated group, GSH:glutathione, GSSG:glutathione disulfide. Values are mean ±SEM (*p<0.05 vs control **p<0.01 vs control †p<0.05 vs angiotensin II ††p<0.01 vs angiotensin II).

### Plasma GSH and Whole Blood GSH/GSSG

Administration of BSO was significantly reduced GSH content in plasma (Table 1). BSO also reduced the level of GSH/GSSG in the whole blood sample. In contrast, AII did not affect the GSH/GSSG level in the whole blood except plasma GSH (Table 1).

### ROS Production in Polymorphonuclear Cells (PMNCs)

**Figure 1:**
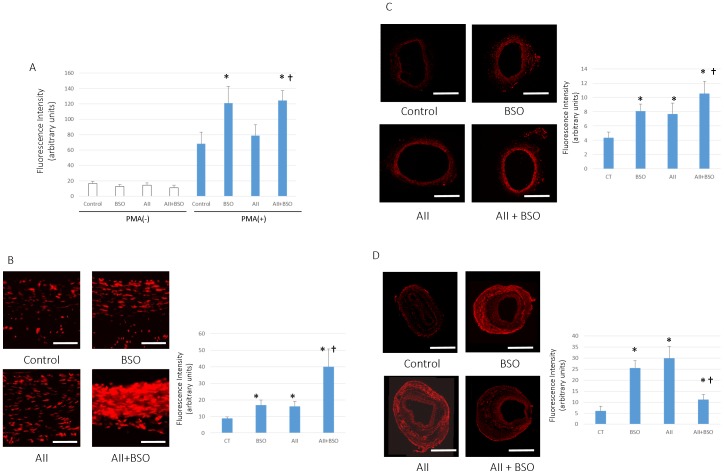
Changes in ROS production. (A) PMA stimulated ROS production in PMNCs. Neutrophils were stimulated by 10 nmol/L PMA and ROS production was measured by Flow-cytometer as the intensity of DCFH fluorescence of 3000 cells. Bar graphs represent mean fluorescent intensity ± SD (*p<0.05 vs control with PMA stimulation, †*p*<0.05 vs AII with PMA stimulation, n = 6–8 for each group). (B–D) Superoxide production in the aortic wall. In situ detection of vascular superoxide production in Aorta (B), femoral arteries without cuff (C) and with cuff injury (D). Tissue superoxide was detected with DHE. Photomicrographs show representative results from 4 separate experiments. Scale bars indicate 50 µm in (B) and 500 µm in (C, D). Bar graphs represent mean fluorescent intensity ± SD (*p<0.05 vs Control, †*p*<0.05 vs BSO and AII, n = 7 of each). All statistical comparisons were evaluated using one-way ANOVA followed by Bonferroni post hoc analysis.

The basal ROS production in PMNCs of each groups were no significant difference (Figure 1A). PMA stimulated ROS production showed 4.18±0.85 fold increment in control group, which was significantly enhanced by BSO. 9.61±2.22 fold compared to the basal level (*p*<0.05). PMA stimulated ROS production was also evident by A II, 5.52±1.11 fold increment from the basal level, however, it was not significantly different from control, PMA stimulation alone. PMA stimulated ROS production of A II pretreated PMNCs was markedly enhanced by co-treatment with BSO, 11.15±1.90 fold (Figure 1A).

### Superoxide Production in the Aortic Wall

To estimate the oxidative stress in vascular wall, we measured the production of superoxide in each arteries by DHE staining. As shown in Figure 1B, 1C. AII or BSO treatment enhanced the production of superoxide in the media of vascular wall. The combination of AII and BSO treatment synergistically enhanced the staining of DHE. In contrast, the production of superoxide in adventitia of cuff injured arteries, was not increased in AII and BSO combination (figure 1D).

### The Effects of GSH Depletion on Vascular Remodeling

**Figure 2:**
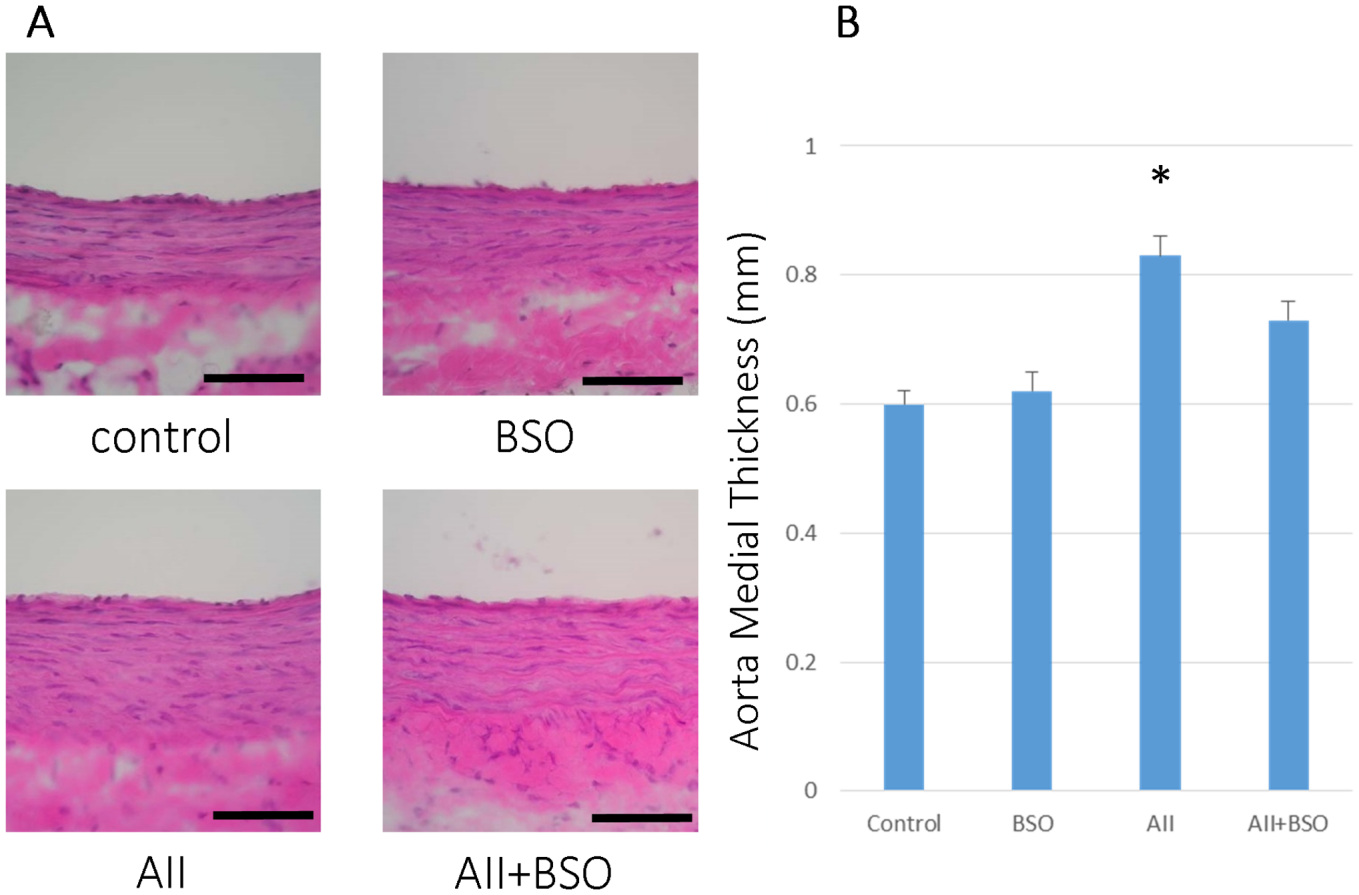
BSO inhibited AII induced aorta medial thickness. (A) Representative micrographs of cross sections of aorta 4 weeks after treatment. Hematoxylin and eosin staining: magnification ×400, Scale bars indicate 500 µm. (B) Graphs shows medial thickness of Aorta. Bar graphs represent mean ± SD (*p<0.05 vs control, n = 7 of each).

**Figure 3:**
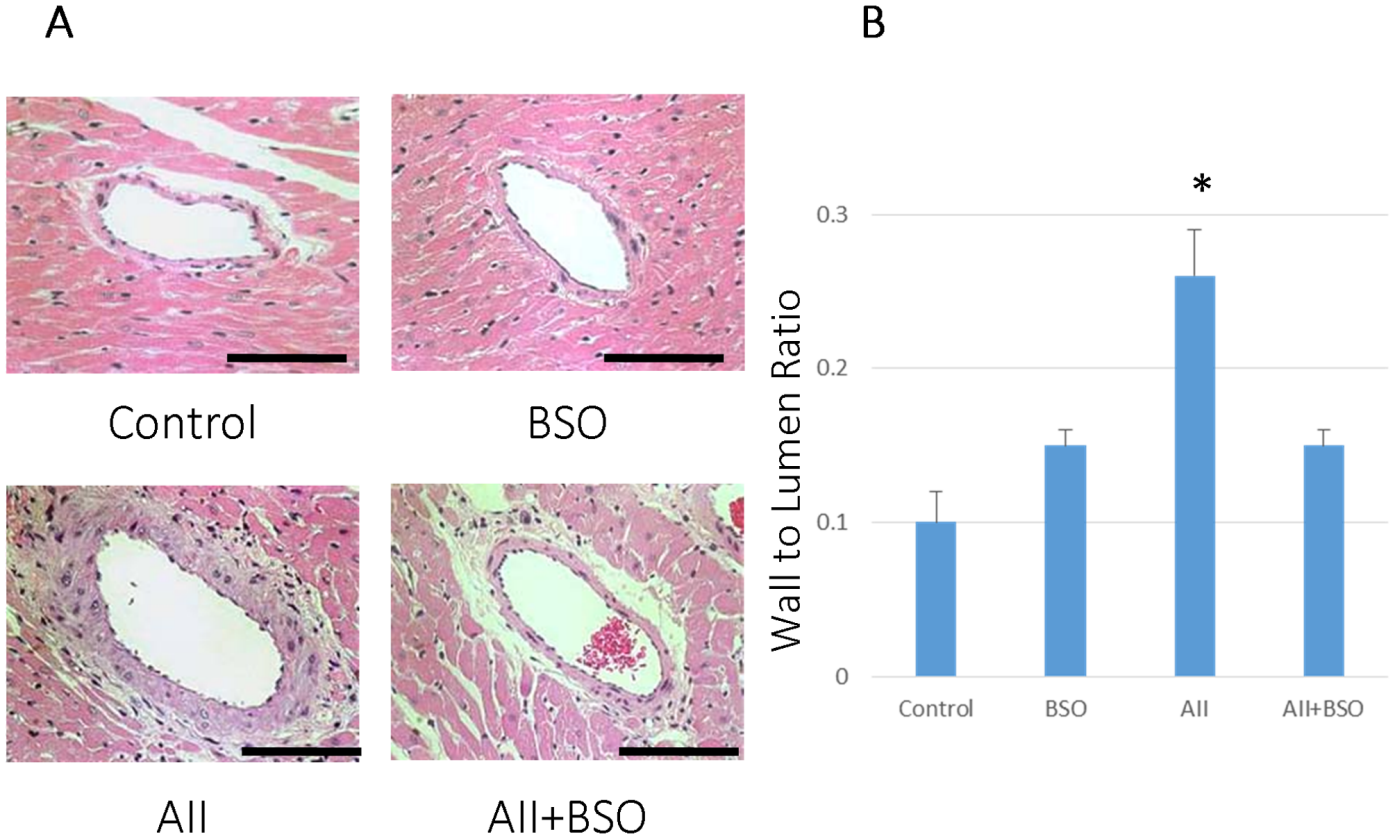
BSO inhibited AII induced medial thickness of coronary artery. (A) The effects of AII and BSO on coronary arteries. Representative micrographs of cross sections of coronary arteries with hematoxylin and eosin staining. : magnification ×400, Bar indicates 100 µm. (B) Graphs shows coronary wall to lumen ratio. Bar graphs represent mean ± SD (*p<0.01 vs AII+BSO, n = 7 of each).

The medial thickness of aortas was not significantly increased by BSO (0.62±0.03 mm) as compared to control (0.60±0.02 mm). However, it was significantly increased by AII (0.83±0.03 mm, *p*<0.01) and the increase was significantly suppressed by the co-treatment with BSO (0.73±0.03 mm, *p*<0.05) (Figure 2). The wall to lumen ratio of coronary arteries was not significantly affected by BSO (0.15±0.01), but was significantly increased by AII (0.10±0.02 mm to 0.26±0.03 mm, *p*<0.01) as well (Figure 3). The increase in the wall to lumen ratio by AII was also suppressed by the co-treatment with BSO (0.15±0.01 mm) (Figure 3).

**Figure 4:**
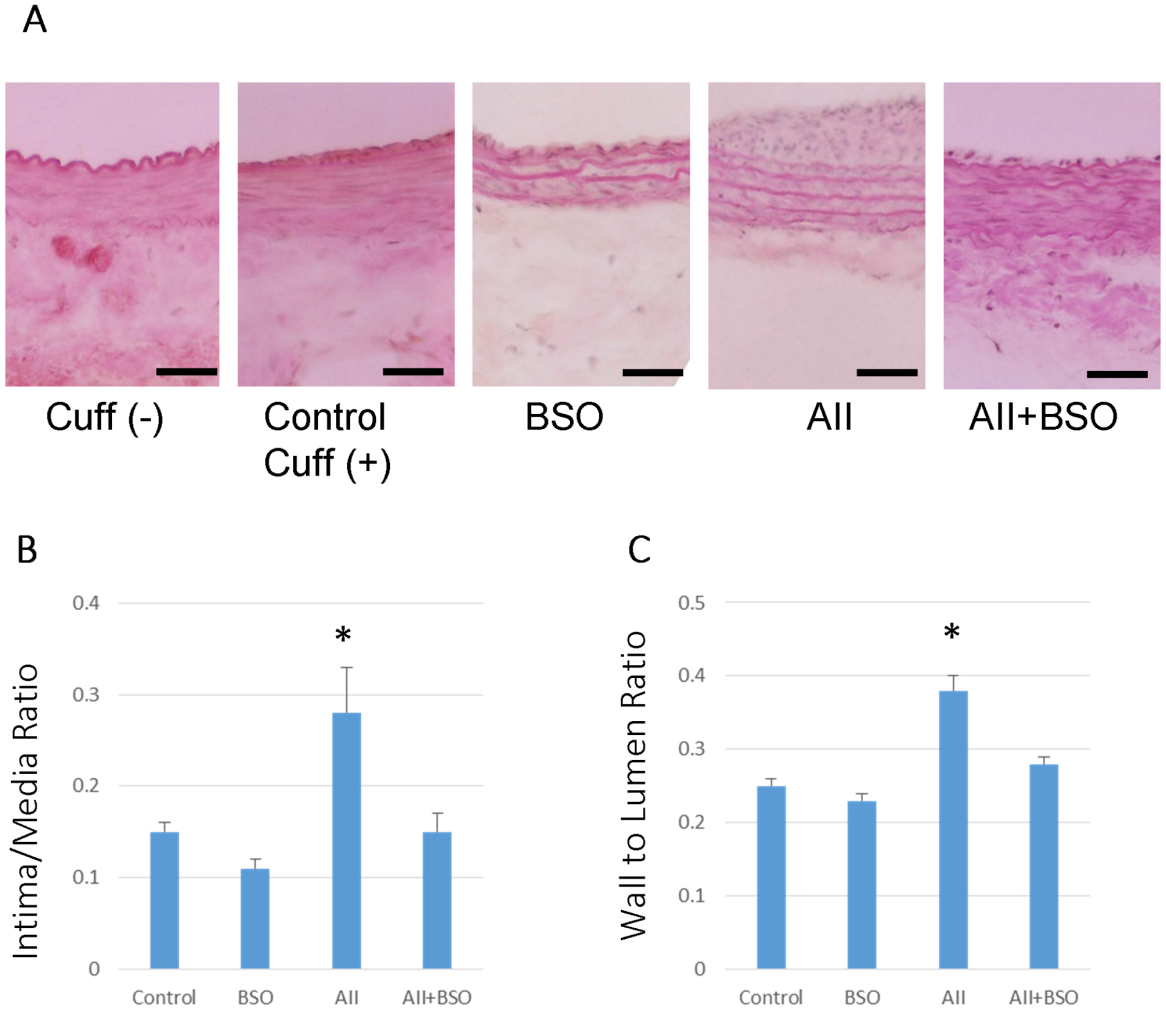
The effects of AII and BSO on neointimal regression in cuff-injured arteries. (A) Representative light micrographs of femoral artery cross sections. 4 weeks after cuff injury, arteries were stained with hematoxylin and eosin: control artery without cuff (Cuff(−)), cuff injured control artery (Control), treated with BSO (BSO), AII (AII), and AII and BSO (AII+BSO). Scale bars indicate 50 µm. Graphs show ratio of intimal area to medial area (I/M ratio) (B) and ratio of wall (media) to lumen (C) of cuff injured artery. Bar graphs represent mean ± SD (*p<0.05 vs AII+BSO, n = 7 of each).

At 4 weeks after cuff injury, an obvious neointimal formation was found in control (Figure 4A), and it was significantly enhanced by AII (Figure 4A, B) (0.15±0.01 to 0.28±0.05 mm in intima/media ratio; *p*<0.05). BSO alone did not affect the neointimal formation (0.11±0.01), but the co-treatment with BSO significantly attenuated AII-induced neointimal formation (0.15±0.02 mm; *p*<0.05, Figure 4B).

The wall to lumen ratio of cuff injured arteries was not significantly affected by BSO (0.23±0.01 mm), but was significantly increased by AII 0.25±0.01 to 0.38±0.02 mm (*p*<0.01) (Figure 4C). The increase in the wall to lumen ratio by AII was significantly suppressed by the co-treatment with BSO (0.28±0.01 mm).

### GSH Depletion Induced Apoptosis in Formed Neointimal Cells

**Figure 5:**
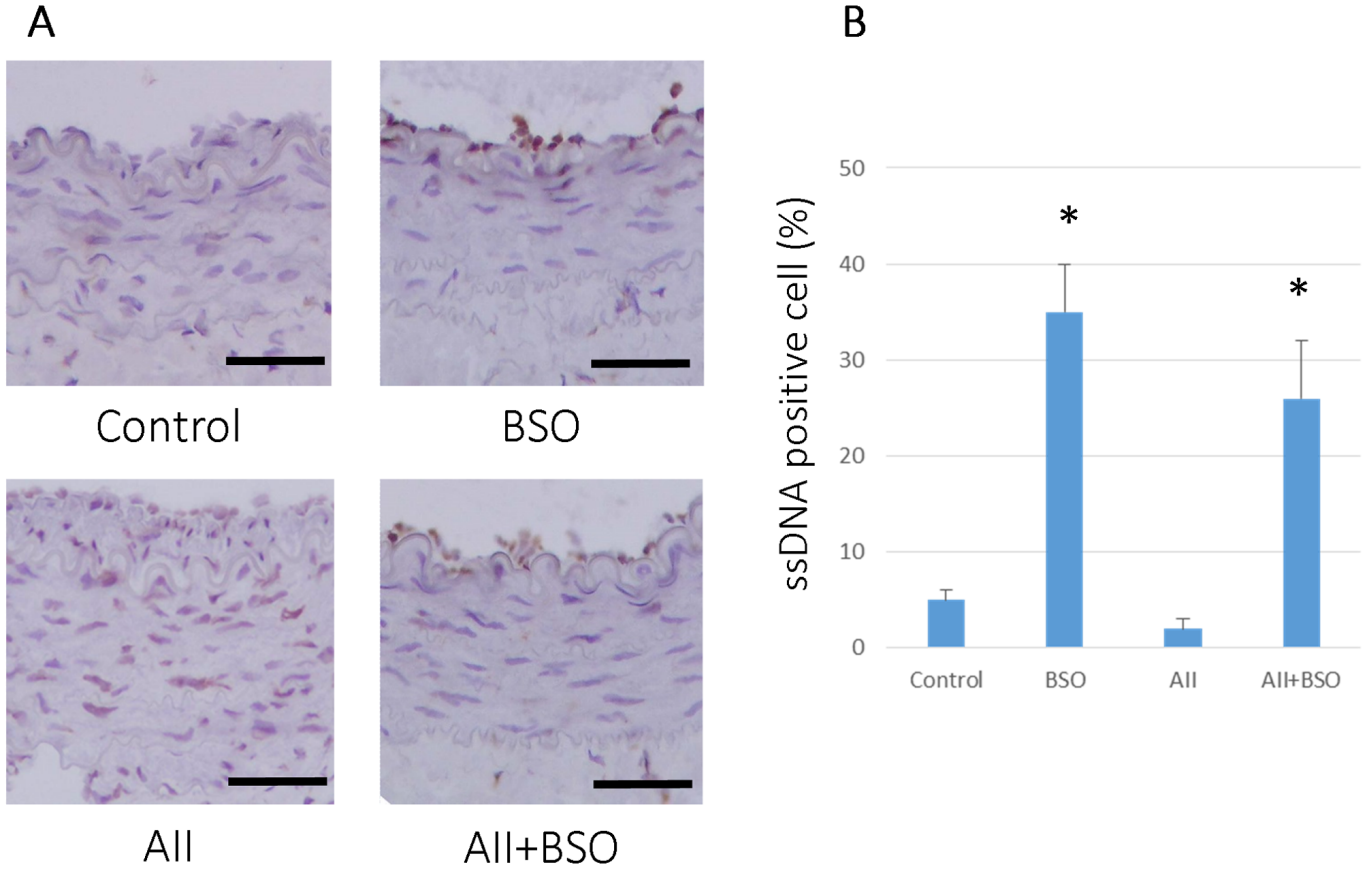
GSH reduction induced apoptosis in cuff-injured arteries. (A) Representative immunohistochemical photomicrographs of femoral artery cross sections. 4 weeks after cuff injury, arteries were stained with anti-single strand DNA antibody: cuff injured control artery (Control), treated with BSO (BSO), AII (AII), and AII and BSO (AII+BSO). Reactions were stained with horseradish peroxidase substrates for color development, with diaminobenzidine in brown. Nuclei were counterstained with hematoxylin. Scale bars indicate 50 µm. (B) Graph shows ratio of apoptotic cells in intima at 4 weeks after cuff injury. Bar graphs represent mean ± SD (*p<0.01 vs AII, n = 7 of each).

To determine the effects of BSO on AII-induced vascular remodeling, we measured the apoptotic change and cell proliferation in vivo. As shown in Figure 5, BSO treatment induced apoptotic cells (ssDNA positive cells) in neointima compared to control (0.05±0.01 to 0.35±0.05%, *p*<0.01). AII did not affect the number of ssDNA positive cells (0.02±0.01%). In contrast, the co-treatment with BSO significantly increased the number of ssDNA positive cells, 0.26±0.06% (*p*<0.01). There were also no statistical difference in PCNA positive cells after 4-weeks-treatment (data not shown).

### GSH Depletion Reduced AII induced VSMCs proliferation

We preliminary investigated the changes in GSH content of VSMCs with BSO treatment. Treatment with BSO caused a dose-dependent decrease in cellular GSH levels, and was toxic in LDH assay at concentrations over 1 mmol/L, 36 hours (data not shown).

**Figure 6:**
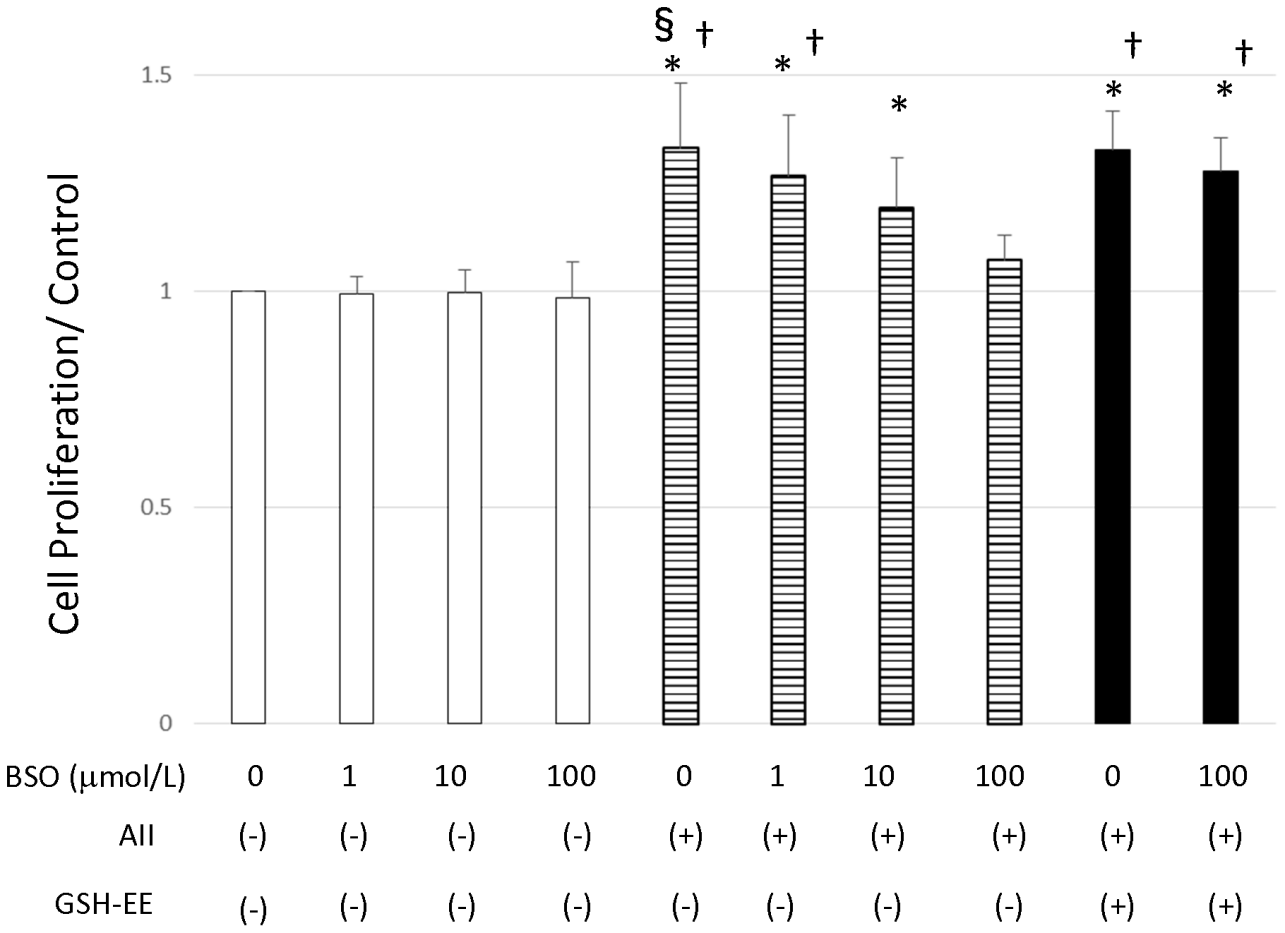
GSH reduction reduced AII induced VSMC proliferation. VSMCs proliferation were measured by BrdU cell proliferation assay. Values of non-treated control group were set at 100%. Bar graphs represent mean ± SD (*p<0.01 vs control (BSO(−), AII(−) and GSE-EE(−)), †*p*<0.01 vs BSO100, AII(+) and GSH-EE(−) group, §*p*<0.05 vs BSO10, AII and GSH-EE(−) group respectively, n = 4 of each, Data was measured in triplicate). AII: AII, GSH-EE: Glutathione ethyl ester.

AII (0.1 µmol/L) significantly increased VSMC proliferation (Figure 6). BSO alone did not affect VSMC proliferation even at the maximum dose 100 µmol/L (Figure 6). However, BSO suppressed AII-induced VSMC proliferation, dose-dependently. The anti-proliferative effects of BSO were reversed by 1 mmol/L glutathione ethyl ester. Glutathione ethyl ester itself did not affect VSMCs proliferation.

### Circumferential Vascular Wall Stress

Values were calculated using Laplace theorem from blood pressure, lumen radius and wall thickness. At 4weeks after cuff injury, systolic blood pressure of control, AII, BSO and AII+BSO was 110.9±6.4, 214.6±19.0, 115±21.4 and 212.4 ±25.3 mmHg, respectively. Mean blood pressure of control, AII, BSO and AII+BSO was 93.5±8.3, 156.3±28.6, 99.3±5.1 and 147.7±23.2 mmHg, respectively.

**Figure 7:**
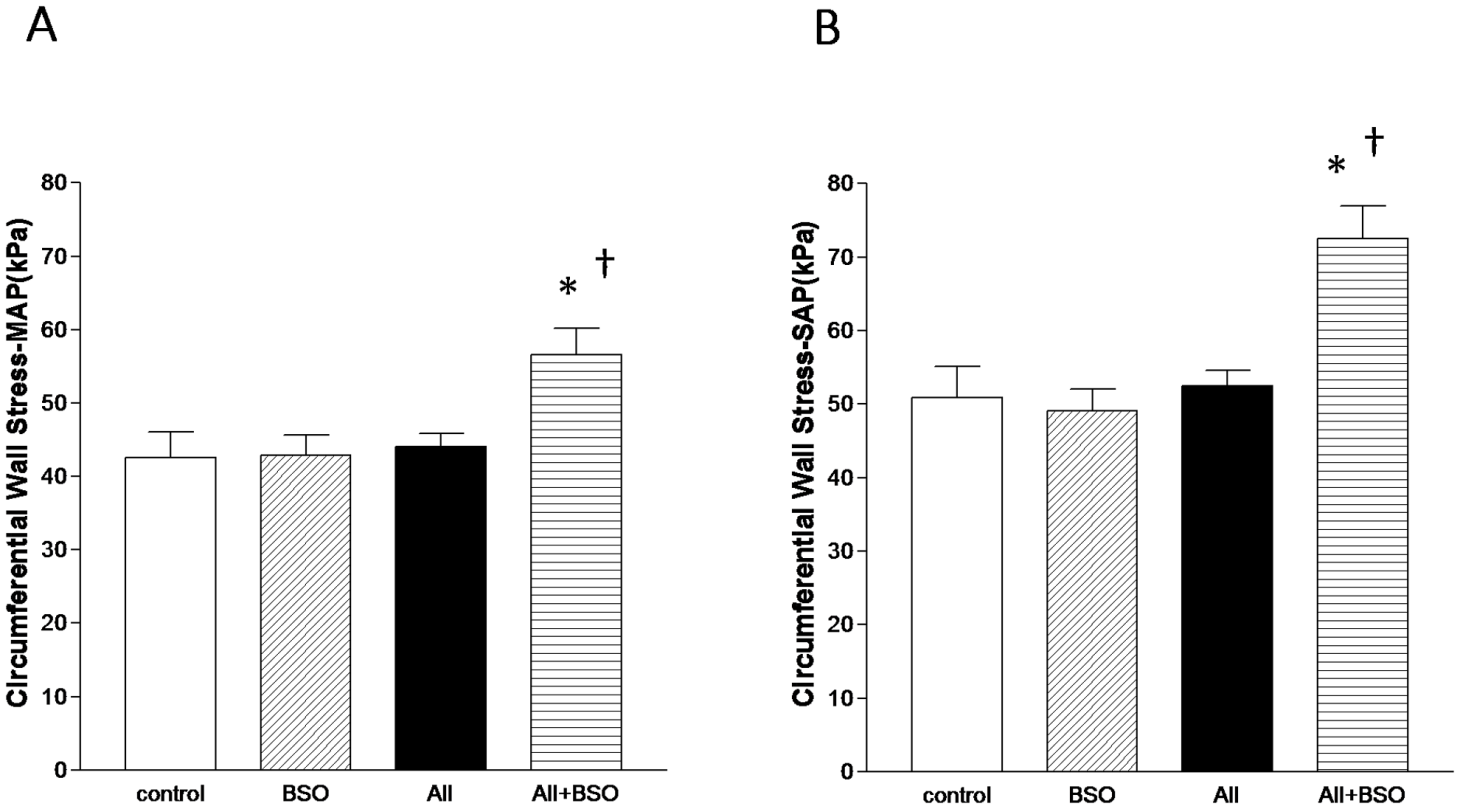
Circumferential Vascular Wall Stresses. Graphs shows mean circumferential vascular wall stress (σ_cMAP_) (A) and systolic circumferential vascular wall stress (σ_cSAP_) (B). Values were calculated using Laplace theorem from blood pressure, lumen radius and wall thickness. Bar graph represent mean ± SD (*p<0.01 vs BSO, †*p*<0.01 vs AII, n = 7 of each).

The calculated mean (σ_cMAP_) (Figure 7A) and systolic circumferential vascular wall stresses (σ_cSAP_) (Figure 7B) were not affected by BSO (44.1±1.8, 52.5±2.1 kPa) compared to control (42.5±3.6, 50.9±4.2 kPa). Despite of elevated blood pressure, σ_cMAP_ and σ_cSAP_ were well compensated in AII (42.9±2.7, 49.0±3.0 kPa). In contrast, both σ_cMAP_ and σ_cSAP_ were significantly increased in AII with BSO (56.6±3.6, *p*<0.05, 72.5±4.5 kPa, *p*<0.01) (Figure 7A, 7B).

### Localization of gp91-phox

**Figure 8:**
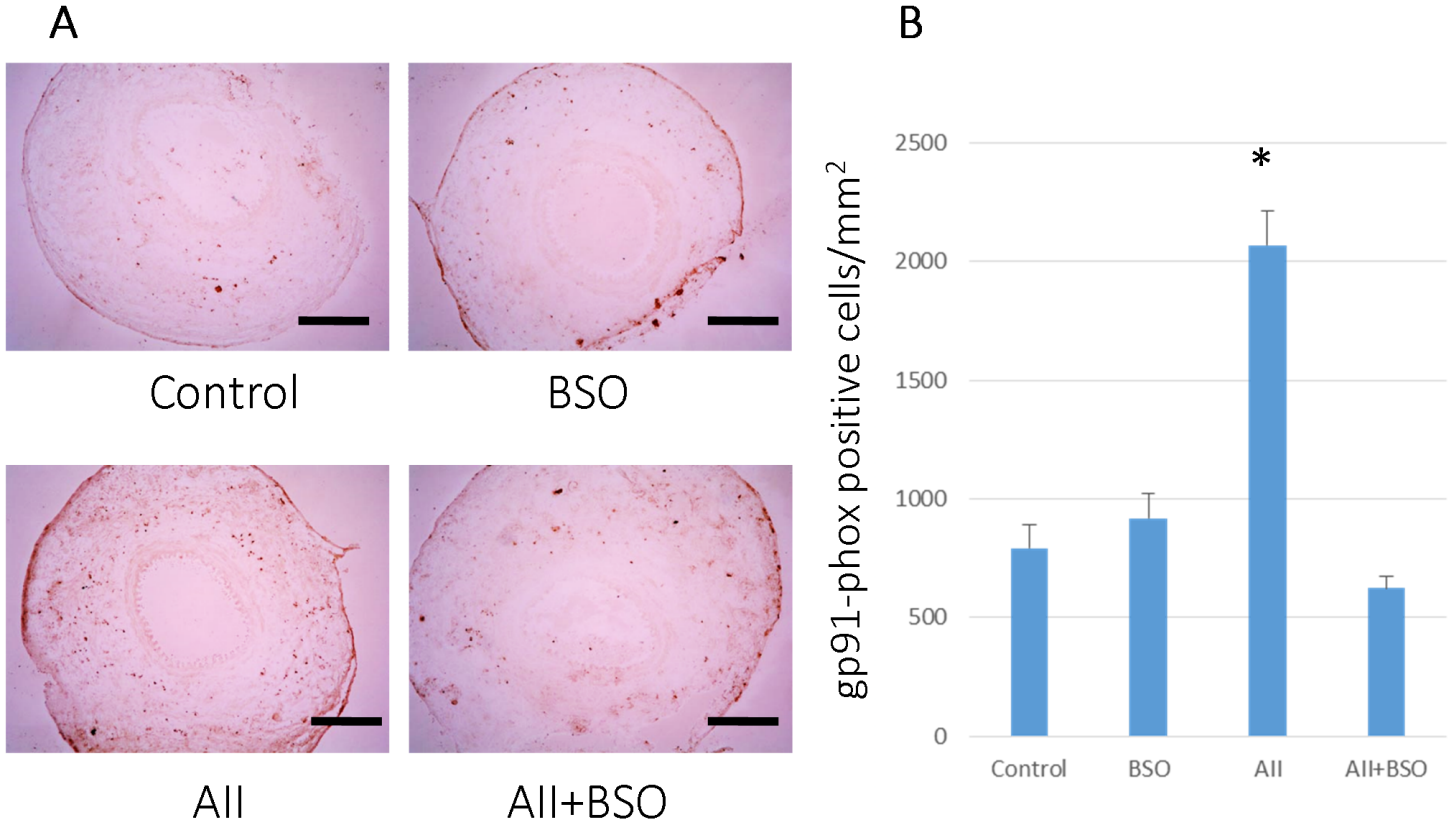
Localization of gp91-phox in cuff-injured arteries. (A) Representative immunohistochemical photomicrographs of femoral artery cross sections. 1 weeks after cuff injury, arteries were stained with anti-gp91 phox antibody: cuff injured control artery (Control), treated with BSO (BSO), AII (AII), and AII and BSO (AII+BSO). Reactions were stained with horseradish peroxidase substrates for color development, with diaminobenzidine in brown. Scale bars indicate 200 µm. (B) Graph shows ratio of apoptotic cells in intima at 4 weeks after cuff injury. Bar graphs represent mean ± SD (*p<0.01 vs AII+BSO, n = 5 of each).

At 1 week after cuff injury, gp91-phox, one of the major component of NADPH oxidase, was detected in adventitia of cuff injured arteries (Figure 8) (792.8±97.1/mm^2^). Although gp91-phox positive cells did not show remarkable increase with BSO alone (918.7±104.6/mm^2^), markedly increased with AII treatment (2068.5±148.2/mm^2^, p<0.01). In contrast, the co-treatment with BSO were significantly reduced the number of gp91-phox positive cells (622.7±52.2/mm^2^, *p*<0.01).

## Discussion

This study demonstrated for the first time that AII-induced vascular, not cardiac remodeling was almost totally abolished by enhanced oxidative stress in impaired GSH redox state.

We employed BSO, a GSH synthase inhibitor, to enhance oxidative stress in rats and cultured VSMCs. GSH is one of the major components of the antioxidant defense system, participates in the reduction of disulfides and other molecules, and protects cells against the destructive effects of ROS and free radicals [10], [13]. BSO 30 mmol/L was the same dose used in the previous in vivo studies, which were sufficiently high to deplete GSH and enhance oxidative stress [11], [12]. We confirmed a decreased GSH/GSSG content in whole blood and plasma of BSO treated rats. We also confirmed an increased capacity of ROS production in PMNCs and an increased superoxide production in vascular wall indicating increased oxidative stress by BSO-treatment. As we expected, AII-induced superoxide production in vascular wall was additively enhanced by BSO treatment. However, surprisingly, the vascular remodeling induced by AII was oppositely suppressed.

The increased intima/media ratio indicating neointimal formation, and the increased wall to lumen ratio indicating medial hyperplasia were two major hallmarks of AII induced enhancement of vascular remodeling in cuff injured artery [8], [27]. In fact, our results also showed neointimal formation and medial hypertrophy in AII treatment (Figure 4). Both AII-induced neointimal formation and medial thickening were attenuated by enhanced oxidative stress of impaired GSH redox, but the mechanisms related to each remodeling process may be different. The former may be induced by enhancement of apoptosis in the neointima (Figure 5), and the later may be based on insufficient medial hypertrophy (Figure 4A).

Cuff injury produces a diffuse intimal thickness of artery that is similar to early lesions of atherosclerosis [17]. Huth et al. [28] speculated that medial and adventitial hypoxia induced by closing of the vasa vasorum triggered the atherosclerotic process in this model, however, the exact mechanism of intimal thickness in this model remains unclear. Tanaka K *et al*. reported that in neointimal thickening by cuff-injury, bone marrow derived cells are not included in the component in comparison with other blood vessel injury models such as ligation and wire injury [28]. Using the similar cuff injured model of AII type 1 receptor (AT1) and type 2 receptor (AT2) knockout mice, Suzuki J et al demonstrated antiproliferative and proapoptotic effects of AT2, which were counteracted by proliferative and antiapoptotic effects of AT1 in the process of neointimal formation, indicating balanced regulation of apoptosis by AII is necessary in wild type mice [8]. In fact, in the present study, AII, per se, did not affect apoptotic cell rate in the neointima. In contrast, BSO treatment significantly induced apoptosis and markedly enhanced AII induced apoptosis in the neointima. Induction of apoptosis in impaired GSH redox state has been reported in several types of cells and conditions [29]–[32]. Depletion of intracellular GSH by BSO significantly enhanced apoptosis in cultured bovine endothelial cells [30], and reversed antiapoptotic effect of physiological shear stress [31] in cultured human endothelial cells. Redox-sensitive apoptosis of vascular cells was significantly potentiated by depletion of intracellular GSH in photodynamic therapy [32]. The present study demonstrated for the first time impairment of GSH redox system enhanced apoptosis in neointima *in vivo*. Enhanced apoptosis reduced cell numbers in neointima, consequently decreased intimal thickness. We believe enhanced apoptosis is the major mechanism of suppressed vascular remodeling in neointima of cuff injured arteries.

Suppression of medial hyperplasia was another major finding in impaired GSH redox status. AII significantly enhanced medial hyperplasia, and increased media to lumen ratio. Numerous studies have demonstrated that AII which stimulates VSMCs and activates protein synthesis is major factor of hyperplasia and hypertrophy, consequently promotes vascular remodeling [33], [34] Enhanced oxidative stress in impaired GSH redox status induced by BSO has been reported to inhibit proliferation of VSMCs, particularly in the condition with accelerated proliferation [35]. We demonstrated that BSO suppressed AII-induced enhancement of VSMCs proliferation in a dose dependent manner. In contrast, it has been reported that small dosage of BSO enhanced cell proliferation in vitro [36], [37]. It was not surprising because oxidative stress demonstrates dual action depending on the power of stress [38], [39]. Importantly, BSO treatment did not affect basal BrdU incorporation, indicating no toxic effects of depletion of GSH on basal cell proliferation. The higher dose of BSO more than 10 µmol/L, which was comparable to the plasma concentration achieved *in vivo* in the present study, significantly suppressed VSMCs proliferation stimulated by AII. Importantly, suppression of AII-induced proliferation of VSMCs was completely reversed by supplementation of GSH monoethyl ester, indicating the major mechanism is fully GSH dependent.

We demonstrated that the manifestation of AII-induced vascular remodeling was totally suppressed by enhanced oxidative stress of impaired GSH redox status. Suppression of neointimal thickness of cuff injured arteries has some beneficial effects in clinical aspects, similar to atherosclerotic lesions. In contrast, the suppression of medial hyperplasia does not seem to be simply beneficial. The circumferential vascular wall stress was well maintained against high blood pressure by AII, indicating adaptive response of vascular wall. It is noteworthy that AII and enhanced oxidative stress have been recognized to play major roles in aneurismal formation in human [40], [41] as well as in experimental conditions [42]–[44]. The marked suppression of medial hyperplasia caused a thinner vascular wall, which was imbalance with significantly increased blood pressure by AII, consequently increased a circumferential wall stress. This may cause a maladaptive state of the vasculature which is intolerable to the mechanical stress of high blood pressure.

Morphological changes in coronary vasculatures are recognized as an important adaptive process in pressure overload-induced left ventricular hypertrophy (LVH) [45]. In the present study, we found that BSO significantly suppressed medial hyperplasia in coronary arteries similar to cuff injured femoral arteries, but not altered LVH. This may indicates LVH is derived mainly from mechanical stress of high blood pressure, and is not easily affected by markedly enhanced oxidative stress in impaired GSH redox status. Further study is required to clarify these differences in the mechanisms of modification of cardiovascular remodeling.

In conclusions, a vast oxidative stress in impaired GSH redox system totally abolished AII-induced vascular remodeling via enhancement of apoptosis in the neointima and suppression of cell growth in the media. A drastic suppression of remodeling, however, increased vascular wall stress, which was imbalance with marked hypertension, may be resulting in “maladaptive vasculature” intolerable to mechanical stress of AII.
